# Uterine Arteries Resistance in Pregnant Women with Gestational Diabetes Mellitus, Diabetes Mellitus Type 1, Diabetes Mellitus Type 2, and Uncomplicated Pregnancies

**DOI:** 10.3390/biomedicines11123106

**Published:** 2023-11-21

**Authors:** Christos Chatzakis, Makarios Eleftheriades, Eleftheria Demertzidou, Anna Eleftheriades, Nikolaos Koletsos, Lazaros Lavasidis, Athanasios Zikopoulos, Konstantinos Dinas, Alexandros Sotiriadis

**Affiliations:** 1 Second Department of Obstetrics and Gynecology, School of Medicine, Aristotle University of Thessaloniki, 546 42 Thessaloniki, Greece; cchatzak@auth.gr (C.C.);; 2 Second Department of Obstetrics and Gynecology, Medical School, National and Capodistrian University of Athens, 115 28 Athens, Greece; makarios@hotmail.co.uk (M.E.); annelefth@gmail.com (A.E.); 3 Department of Rheumatology, University of Ioannina, 451 10 Ioannina, Greece; nick.koletsos@gmail.com; 4 Obstetrics and Gynecology Royal Cornwall Hospital, Truro TR1 3LQ, UK

**Keywords:** uterine arteries, pre-existing diabetes, diabetes mellitus type 1, diabetes mellitus type 2, gestational diabetes mellitus

## Abstract

Background: The examination of the uterine arteries using Doppler in the first trimester of pregnancy serves as a valuable tool for evaluating the uteroplacental circulation. Diabetes mellitus is associated with altered placental implantation and pregnancy-related pathologies, such as preeclampsia. The aim of this study was to compare the uterine arteries’ pulsatility indices (UtA PI) in women with diabetes mellitus type 1 (DM1), diabetes mellitus type 2 (DM2), gestational diabetes mellitus (GDM), and uncomplicated pregnancies. Methods: This was a retrospective case–control trial including pregnant women with DM1, DM2, GDM, and uncomplicated pregnancies, presenting for first-trimester ultrasound screening in two tertiary university hospitals between 2013 and 2023. The first-trimester UtA pulsatility index (PI), expressed in multiples of medians (MoMs), was compared between the four groups. Results: Out of 15,638 pregnant women, 58 women with DM1, 67 women with DM2, 65 women with GDM, and 65 women with uncomplicated pregnancies were included. The mean UtA PI were 1.00 ± 0.26 MoMs, 1.04 ± 0.32 MoMs, 1.02 ± 0.31 MoMs, and 1.08 ± 0.33 MoMs in pregnant women with DM1, DM2, GDM, and uncomplicated pregnancies, respectively (*p* > 0.05). Conclusions: Potential alterations in the implantation of the placenta in pregnant women with diabetes were not displayed in the first-trimester pulsatility indices of the uterine arteries, as there were no changes between the groups.

## 1. Introduction

The intricate interplay between the uterus and the placenta plays a pivotal role in the development of pregnancy [1]. The invading trophoblast, a specialized cell type, allows for a transformation of the small, tightly coiled spiral arteries of the uterus into large, dilated vessels [2]. This transformation is essential to ensuring a sufficient blood supply to the developing fetus [3,4]. As such, the examination of the uterine arteries using Doppler techniques serves as a valuable tool for evaluating the circulation at the uteroplacental interface, especially during the critical first trimester of pregnancy [5].

This assessment becomes particularly relevant in instances where the trophoblast fails to invade the uterine spiral arteries successfully, resulting in the retention of smooth muscle and elastin within these arteries [6]. This phenomenon of an unfavorable environment is commonly referred to as defective implantation, and it can have significant implications for the health of both the mother and the developing fetus [6,7]. In cases of defective implantation, the expected transformation of the uteroplacental circulation into a system characterized by low resistance and high capacitance does not occur as it should [8]. The resistance to blood flow at the uteroplacental interface is then transmitted in an upstream direction to the uterine arteries, ultimately leading to an elevated pulsatility index (PI) [9].

Diabetes mellitus (DM) is a condition that can significantly impact the process of placental implantation during pregnancy [10]. Individuals with diabetes, particularly those with poorly managed pre-existing diabetes or gestational diabetes, present with an increased risk of complications that can negatively affect the attachment of the placenta to the endometrium [11,12]. High blood glucose levels characteristic of diabetes can lead to vascular changes, enhanced inflammation processes, and increased oxidative stress. These changes can subsequently impair the placental blood flow, creating an adverse uteroplacental environment [13]. Such abnormalities in the uteroplacental environment can result in severe complications such as preeclampsia and fetal growth restriction, and can have an overall impact on maternal–fetal well-being [14,15].

The purpose of the current study was to investigate whether the uterine arteries of patients with diabetes mellitus type 1, type 2, or gestational would show early alterations compared to pregnant women without diabetes. Therefore, the uterine arteries’ pulsatility indices (PIs) were measured during the first trimester of pregnancy and expressed in multiples of medians (MoMs). The findings of this research aim to shed light on potential early indicators and alterations in uterine artery function related to diabetes, contributing to a better understanding of the intricate dynamics of pregnancy in diabetic individuals and potentially aiding in improving clinical management and outcomes.

## 2. Materials and Methods

### 2.1. Study Design

The current study was a retrospective case–control trial.

### 2.2. Setting

The study included pregnant women with diabetes mellitus type 1 (DM1), diabetes mellitus type 2 (DM2), gestational diabetes mellitus (GDM), and without any type of diabetes. The participants visited the obstetric outpatient departments of the second department of Obstetrics and Gynecology of Aristotle University of Thessaloniki and the second department of Obstetrics and Gynecology of National and Kapodistrian University of Athens, and underwent first-trimester ultrasound examination, between 2013 and 2023. All participating women signed a consent form stating that their anonymized data could be used for research and auditing purposes. The current study was approved by the ethical committee and the institutional review board of the Areteion Hospital, and is in accordance with the declaration of Helsinki.

### 2.3. Participants

The participants in this study were derived from the population of pregnant women who presented to the outpatient departments in order to undergo the first-trimester combined screening test for aneuploidies. Women with DM1 and DM2, without diabetes complications (proliferative retinopathy, nephropathy, neuropathy, and hypertension), were included in the study. Subsequently, pregnant women with uncomplicated pregnancies and pregnant women with GDM were randomly (using the open-source software R 2.15.1, the R Foundation for Statistical Computing) selected and included in the study. Women with DM1 and DM2 were included if they presented with a definitive diagnosis by the time they became pregnant and underwent continuous evaluations by an endocrinologist. Women with GDM were diagnosed as per the International Association of Diabetes and Pregnancy Study Groups (IADPSG) criteria after having an abnormal oral glucose tolerance test (OGTT) between 24 and 28 weeks of pregnancy [16]. Pathological OGTT is defined as having either a fasting glucose level equal to or greater than 92 mg/dL; a post-prandial glucose level equal to or greater than 180 mg/dL at 1 h, or equal to or greater than 153 mg/dL at 2 h after consumption of a glucose solution; or any combination of all three criteria.

### 2.4. Variables

The outcome of interest was the pulsatility index (PI) of the uterine arteries, expressed in multiples of medians (MoMs), in the first trimester of pregnancy (Figure S1).

All ultrasound examinations were performed by sonographers certified by the Fetal Medicine Foundation (www.fetalmedicine.com, 18 October 2023), and dating of the pregnancy was based on the crown–rump length (CRL, according to the International Society of Ultrasound in Obstetrics and Gynecology (ISUOG) guideline for first-trimester screening ultrasounds [17]. All ultrasound findings were recorded. The maternal and pregnancy characteristics which were recorded included the maternal age, maternal BMI, medical history, smoking status, parity, and method of conception. All of the data were recorded using Astraia software 1.2 (Astraia GmbH, Munich, Germany).

### 2.5. Statistical Methods

In this study, the normality of the distribution for continuous variables was rigorously assessed to determine the appropriate statistical tests and presentation methods. Two common methods were typically employed for assessing the normality of data: visual inspection and statistical tests. Visual methods, such as histograms, box plots, and quantile–quantile (Q-Q) plots, were used to examine the shape of the data distribution. A histogram provides a graphical representation of the data’s frequency distribution, while a box plot visualizes the spread and central tendency of the data. Q-Q plots compare the data’s quantiles to those of a theoretical normal distribution. Deviations from the expected patterns in these visual representations can be indicative of non-normality. On the other hand, several statistical tests are available to quantitatively assess the normality of data. The Shapiro–Wilk test and the Kolmogorov–Smirnov test are common tools used for this purpose. These tests provide *p*-values that indicate whether the data significantly deviate from a normal distribution. A low *p*-value suggests non-normality, while a high *p*-value suggests that the data follow a normal distribution. Based on the results of these assessments, it was determined whether continuous variables could be appropriately described using the means and standard deviations for normally distributed data, or the medians and interquartile range (IQR) values for non-normally distributed data. This dual presentation approach allowed us to create accurate representation of the data’s central tendency and spread, considering the inherent characteristics of the variables in question. Categorical variables, on the other hand, were succinctly summarized in terms of percentages, enabling a clear representation of their prevalence within each group.

To explore the differences among the four study groups, various statistical tests were employed. Continuous variables underwent analysis using ANOVA when they met the assumption of normal distribution, whereas the Kruskal–Wallis test was used for non-normally distributed variables. Subsequently, Bonferroni tests were conducted for post-hoc analysis, aiding in the identification of specific group differences. For the assessment of proportions within the categorical variables, Chi-square or Fisher’s exact tests were chosen based on the appropriateness of the statistical method. Additionally, odds ratios (ORs) and their corresponding 95% confidence intervals (CIs) were computed to further investigate and quantify the associations between variables.

In all statistical tests which we conducted, a significance level of <0.05 was employed as the threshold for determining the presence of significant differences or associations. The adoption of this significance level ensured the robustness of the findings and helped to establish the credibility of the results.

Furthermore, linear regression analysis was employed to examine the potential predictors of the uterine arteries’ pulsatility indices’ (PIs) multiples of medians (MoMs). This analysis integrated maternal characteristics and clinical findings as potential predictors, accounting for any potential confounding variables that might have influenced the outcome. This comprehensive analytical approach aimed to unravel the intricate relationships between these variables and uterine artery PI MoMs. Importantly, all statistical analyses were carried out using open-source software, specifically R 2.15.1, developed by The R Foundation for Statistical Computing. This choice of software ensured transparency and accessibility in the statistical analysis process, enabling researchers to replicate and build upon the study’s findings.

## 3. Results

### 3.1. Participants

The initial cohort consisted of 15,638 pregnancies. There were 58 women with DM1 and 67 with DM2 in the first trimester, whilst 2024 women developed GDM later in pregnancy. Of the pregnant women with the uncomplicated pregnancies and the pregnant women with GDM, 65 women were randomly selected from each group.

### 3.2. Descriptive Data

The maternal characteristics and clinical findings of the included women, according to the four different groups, are presented in Table 1. Several key variables were assessed, shedding light on their potential impacts on the outcomes of interest. Firstly, the mean body mass index (BMI) values in the four groups showed no significant differences, as indicated by the ANOVA test (*p* > 0.05). This suggests that, at the onset of pregnancy, the groups had similar weight profiles, with no significant variations in average BMI. Nulliparity, a critical factor in pregnancy outcomes, was also explored. The study revealed that the nulliparity percentages did not differ significantly between the groups (*p* > 0.05). This indicates that the likelihood of a first-time pregnancy did not significantly vary among individuals with uncomplicated pregnancies and those with different types of diabetes. Likewise, smoking habits during pregnancy were not significantly different across the groups (*p* > 0.05). It is important to note that smoking during pregnancy can have adverse effects, but this study’s findings suggest that, in this sample, smoking habits were fairly consistent among the groups. However, the use of assisted reproductive technology (ART) for conception showed significant differences among the groups (*p* = 0.01). This difference highlights a noteworthy finding—individuals with type 2 diabetes were more likely to have utilized ART for conception when compared to the other groups. Maternal age also emerged as a significant variable in this study, as indicated by the ANOVA test (*p* = 0.001). Subsequent post hoc tests, employing the Bonferroni method, revealed that individuals in the DM2 group had significantly higher maternal ages compared to those in the uncomplicated group (*p* < 0.001) and the GDM group (*p* < 0.001). This suggests that women with type 2 diabetes tend to be slightly older on average when entering pregnancy compared to their counterparts with uncomplicated pregnancies or gestational diabetes. In summary, while BMI, nulliparity, and smoking habits showed no significant variations among the groups, the use of ART for conception and maternal age emerged as variables which differed between the groups. More specifically, women with diabetes mellitus type 2 had higher rates of conception using assisted reproduction techniques compared to the rest of the women. In addition, women with diabetes mellitus type 2 were older in comparison to women with uncomplicated pregnancies and women with gestational diabetes mellitus (Table 1).

### 3.3. Main Results

**Figure 1:**
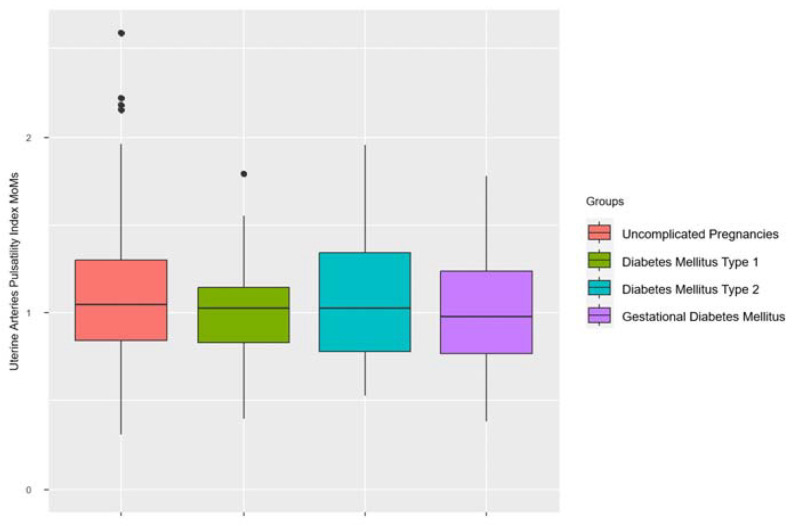
Uterine arteries’ pulsatility indices in multiples of medians in the different groups.

The mean UtA PI was 1.00 ± 0.26 MoMs in women with diabetes mellitus type 1, 1.04 ± 0.32 MoMs in women with diabetes mellitus type 2, 1.02 ± 0.31 MoMs in women with gestational diabetes mellitus, and 1.08 ± 0.33 MoMs in women with uncomplicated pregnancies in the first trimester of pregnancy. The differences between the groups were not statistically significant (*p* > 0.05) (Figure 1).

The independent contributions of the different pathologies (DM1, DM2, and GDM) to the UtA PI MoMs were tested using linear regression analysis, including the maternal age, the maternal BMI, the smoking status, the parity, and the method of conception as potential confounders. However, no variable was found to affect the UtA PI MoMs.

## 4. Discussion

### 4.1. Main Findings

In the current study, the uterine arteries’ pulsatility indices (PIs), expressed in multiples of medians (MoMs), was compared between pregnant women with diabetes mellitus type 1, pregnant women with diabetes mellitus type 2, pregnant women with gestational diabetes mellitus, and pregnant women with uncomplicated pregnancies, and it was found that that there was no statistical difference between these women. Furthermore, an analysis for potential confounders, which included the maternal age, maternal BMI, smoking status, parity, and method of conception, did not reveal any association with the uterine arteries’ pulsatility indices in the first trimester of pregnancy.

### 4.2. Interpretation

The invasion of the trophoblast into the uterine decidua and decidual vessels plays a crucial role in the formation of the placenta [18]. This process ensures that the uterine spiral arteries will transform into a low-resistance and high-capacitance vascular system, which allows for adequate blood volume to be transferred from the mother to the developing pregnancy. This is an essential step in creating a healthy intrauterine environment and ensuring the proper functioning of the placenta, which in turn supports fetal growth [18,19]. This is primarily because maternal blood carries nutrients to the developing fetus and removes waste products. Furthermore, the circulation of blood in the uterine arteries affects the amount of oxygen delivered to the maternal–fetal interface [20,21,22]. Problems in the development of the placenta are linked to the pathogenesis of pregnancy-related pathologies, such as preeclampsia (PE), fetal growth restriction (FGR), and neonates born small for their gestational age (SGA) [14,15]. Preeclampsia engenders a pronounced risk factor for severe complications during pregnancy, including eclampsia, stroke, pulmonary edema, HEELP syndrome, and placental abruption [23,24,25]. Among the nutrients transferred across the placenta is glucose, which is the principal energy provider for placental and fetal metabolism and growth. However, increased maternal blood glucose levels lead to the transportation of glucose through the placenta to the fetus, which results in fetal hyperglycemia and hyperinsulinemia. Increased insulin levels could lead to augmented growth of the fetus [9]. Therefore, the monitoring of changes in the uterine and placental blood vessels starting from the early stages of pregnancy could serve as a valuable diagnostic tool for identifying potential pregnancy complications [26]. Notably, the global prevalence of GDM is 14.7% based on the International Association of Diabetes and Pregnancy Study Groups (IADPSG) criteria, and in the neighborly region, it reaches 30% in high-risk groups of pregnant women [27,28]. Therefore, in the future, we should focus on gestational diabetes, since it is associated not only with adverse pregnancy outcomes, but also with long-term adverse effects on the offspring. This likely occurs due to epigenetic modifications of the fetal genome due to changes in placenta [29].

Quantitative measures can help to identify abnormalities in uterine artery blood flow and recasting, enabling the identification of high-risk pregnant women who may be at greater risk of experiencing adverse pregnancy outcomes [30]. In order to take into account the effects of different factors on the uterine arteries’ pulsatility indices (PIs), Tayyar et al. standardized UtA PI measurements into MoM values using the gestational age, maternal age, maternal weight, maternal race, parity, and previous history of preeclampsia [31]. Therefore, the estimation of the pulsatility indices of the uterine arteries, expressed in multiples of the medians, is recommended as a standard practice for predicting preeclampsia during the first trimester of pregnancy [32,33,34].

Furthermore, in recent years, it has been reported that Doppler examination of the ophthalmic artery has similar predictive value to that of Doppler examination of the uterine arteries for the development of early-onset preeclampsia, and is an accurate and objective technique for assessing maternal hemodynamic circulation [35]. The potential effectiveness of uterine and ophthalmic artery Doppler assessments in preeclampsia screening may be based more on their connection to maternal cardiovascular adjustments during pregnancy than on trophoblast invasion or the transformation of maternal uterine spiral arteries [36]. Moreover, previous studies have indicated that improvements to abnormal uterine artery resistance in preeclamptic women can be achieved with the use of nitric oxide donors, supporting the theory that women with increased uterine artery resistance may have a deficiency of nitric oxide [37]. Thus, the uterine arteries can serve as an indicator, to some extent, of both placental function and endothelial function.

Both pre-existing diabetes mellitus (DM1 and DM2) and gestational diabetes mellitus (GDM) are characterized by a complex interplay of factors, including inflammation, endothelial dysfunction, and oxidative stress [38,39,40,41,42]. All of the aforementioned factors can significantly influence the process of placentation [43,44]. Inflammation is a hallmark of diabetes, leading to a chronic state of low-grade systemic inflammation [45]. This inflammatory environment can adversely affect the placental development processes by disrupting the finely tuned balance of regulatory molecules and cellular interactions required for healthy placentation [46]. Moreover, diabetes has been associated with endothelial dysfunction, which causes predisposition to both macrovascular and microvascular complications. Individuals with DM1, DM2, and GDM have been found to have reduced flow-mediated dilation (FMD), which is the most widely used method to assess endothelial function. It is also interesting that endothelial dysfunction in women with GDM persists in the immediate postpartum period as well [35]. Endothelial dysfunction, a common complication of diabetes, involves impaired blood vessel function, leading to reduced blood flow to various tissues, including the placenta [47,48]. This compromised blood flow can hinder the delivery of vital nutrients and oxygen to the developing fetus, potentially resulting in growth restriction and other complications. Furthermore, oxidative stress, which is characterized by an imbalance between the production of harmful reactive oxygen species (ROS) and the ability of the body to neutralize them, is heightened in diabetes. In diabetic individuals, ROS are produced in various tissues. Thus, excessive ROS can damage placental cells and disrupt their proper functioning, which further exacerbates placental abnormalities [49,50]. Therefore, it can be assumed that the inflammatory milieu, endothelial dysfunction, and oxidative stress, which are associated with diabetes mellitus, collectively contribute to detrimental effects on placentation, posing significant risks to both maternal and fetal health during pregnancy. However, although uterine artery indices are used to detect pathologies associated with abnormal placental implantation, in this study, we demonstrated that the uterine arteries are unable to illustrate the abnormalities in placental implantation which are expected in pregnant women with diabetes mellitus, as there was no difference between women with uncomplicated pregnancies and women with either pre-existing or gestational diabetes mellitus.

The role of diabetes in the development of macrovascular complications such as nephropathy, retinopathy, coronary, cerebrovascular, and peripheral artery disease has been well described; however, it is well known that these complications require time to develop [51]. On the other hand, microvascular complications are apparent even before the manifestation of clinical symptoms [52]. We speculate that altered placental implantation in diabetes is not evident in the uterine arteries, as the uterine arteries are macro-vessels, and longer exposure may be needed for such changes to be manifested.

### 4.3. Strengths and Limitations

This is the first study comparing the resistance of the uterine arteries between pregnant women with diabetes mellitus type 1, diabetes mellitus type 2, gestational diabetes, and uncomplicated pregnancies, attempting to set light to some aspects of the pathogenesis of pregnancy-related complications that are often presented in these patients. Furthermore, the study is in accordance with the STROBE statement case–control studies.

A limitation of this study is that data regarding the glycemic control of the women with diabetes were not available.

## 5. Conclusions

Potential alterations in the placenta implantation in pregnant women with diabetes are not evident in the first-trimester pulsatility indices of uterine arteries, as there were no changes between women with diabetes and women with uncomplicated pregnancies.

## Figures and Tables

**Table 1:** Characteristics of the women included in the different groups.

Groups	Uncomplicated Pregnancies, N = 65	Diabetes Mellitus Type 1, N = 58	Diabetes Mellitus Type 2, N = 67	Gestational Diabetes Mellitus, N = 65	*p* Value
BMI, Mean (SD)	26.1 (5.6)	27.4 (6.8)	29.2 (5.3)	30 (7.4)	>0.05 ^a^
Nulliparity, n (%)	27 (42%)	31 (53%)	29 (43%)	40 (62%)	>0.05 *
Smoking, N (%)	10 (15%)	7 (12%)	9 (13%)	7 (11%)	>0.05 *
ART conception N (%)	1 (1.5%)	3 (5.2%)	12 (18%)	1 (1.5%)	0.01 *
Maternal age, mean (SD)	30 (6.4)	33 (4.1)	34 (5.1)	30 (6, 4)	0.001 ^a^ 0.001 ^b^ <0.001 ^c^

Quantitative variables are presented as means and standard deviations. Comparisons between the groups were carried out using ANOVA and the Bonferroni post hoc test. ^a^ result of ANOVA test; ^b^ post hoc (Bonferroni test), comparing the diabetes mellitus type 2 group with the uncomplicated pregnancies group; and ^c^ post hoc (Bonferroni test), comparing the diabetes mellitus type 2 group and the gestational diabetes mellitus group. Qualitative data are presented in percentages. Chi-square test *.

## Data Availability

Data can be available upon reasonable request, with a thorough description of the proposal for their use, to the corresponding authors.

## Supplementary Materials

The following supporting information can be downloaded at: https://www.mdpi.com/article/10.3390/biomedicines11123106/s1, Strobe checklist. Figure S1: The pulsatility index (PI) of the uterine arteries in the first trimester of pregnancy.
