# Supplementary figures and images for: Uterine Arteries Resistance in Pregnant Women with Gestational Diabetes Mellitus, Diabetes Mellitus Type 1, Diabetes Mellitus Type 2, and Uncomplicated Pregnancies
Source: Biomedicines. 2023 Nov 21;11(12):3106. doi: 10.3390/biomedicines11123106 (PMC10741004; doi:10.3390/biomedicines11123106)

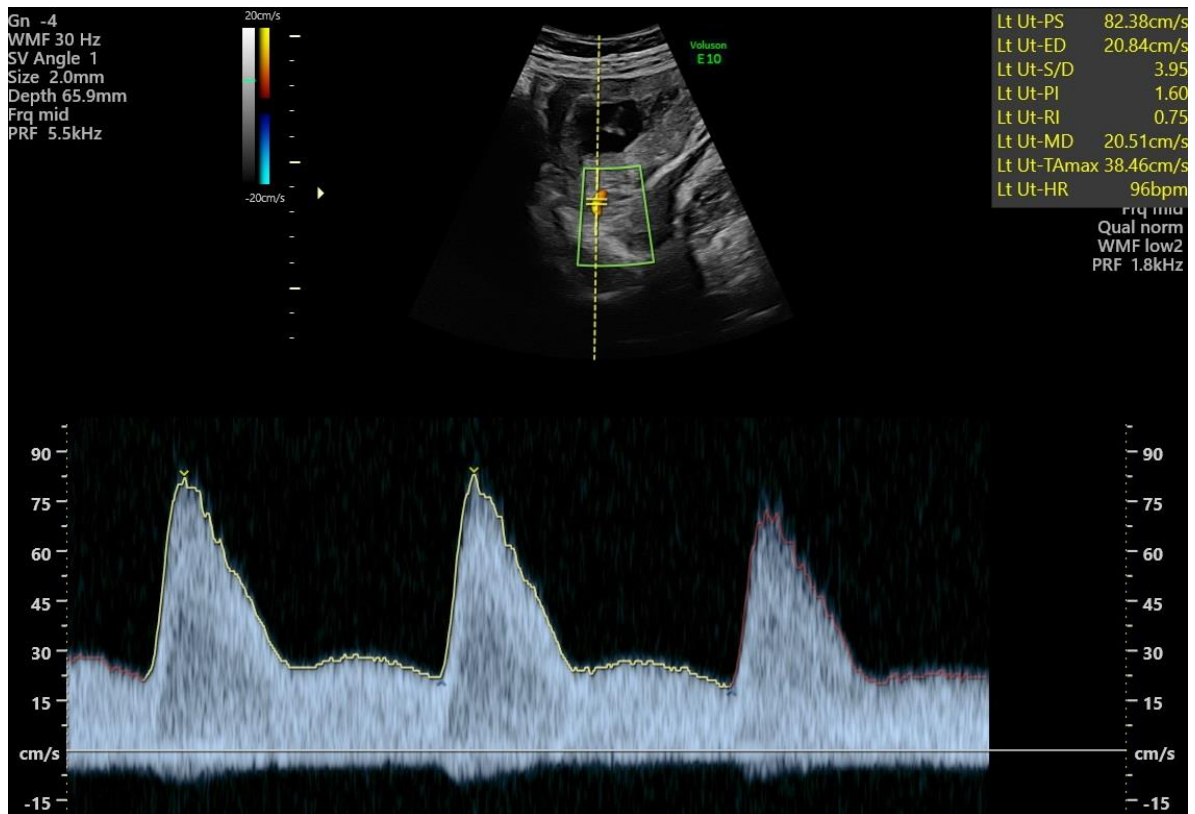

Figure S1. The pulsatility index (PI) of the uterine arteries in the first trimester of pregnancy.

Supplement: Supplementary file 1 [file biomedicines-11-03106-s001.zip › biomedicines-2631070-supplementary.pdf]
